# Dyeing of m-Aramid Fibers in Ionic Liquids

**DOI:** 10.3390/polym12081824

**Published:** 2020-08-14

**Authors:** Klaus Opwis, Bilal Celik, Rainer Benken, Dierk Knittel, Jochen Stefan Gutmann

**Affiliations:** 1 Deutsches Textilforschungszentrum Nord-West gGmbH, Adlerstr. 1, D-47798 Krefeld, Germany; celik@dtnw.de (B.C.); benken@dtnw.de (R.B.); dierk.knittel@gmx.de (D.K.); 2 Department of Physical Chemistry and Center of Nanointegration (CENIDE), University of Duisburg-Essen, Universitaetsstr. 2, 45141 Essen, Germany

**Keywords:** textiles, m-aramid fibers, ionic liquids, dyeing

## Abstract

Aramids represent a class of high-performance fibers with outstanding properties and manifold technical applications, e.g., in flame-retardant protective clothing for firefighters and soldiers. However, the dyeing of aramid fibers is accompanied by several economic and ecological disadvantages, resulting in a high consumption of water, energy and chemicals. In this study, a new and innovative dyeing procedure for m-aramid fibers using ionic liquids (ILs) is presented. The most relevant parameters of IL-dyed fibers, such as tensile strength, elongation and fastness towards washing, rubbing and light, were determined systematically. In summary, all aramid textiles dyed in ILs show similar or even better results than the conventionally dyed samples. In conclusion, we have successfully paved the way for a new, eco-friendly and more sustainable dyeing process for aramids in the near future.

## 1. Introduction

Aramids are polyamids which exhibit aromatic groups in the macromolecular backbone (aromatic polyamids = aramids). The most important species are poly(meta-phenylenterephthalamid) (m-aramid) and poly(para-phenylenterephthalamid) (p-aramid). Both m-aramid and p-aramid are commercialized by the leading producers Teijin (Arnhem, The Netherlands) and DuPont (Wilmington, DE, USA) under the trade names Teijin-conex^®^ and Twaron^®^ and Nomex^®^ and Kevlar^®^, respectively. Aramid fibers excel by their outstanding mechanical and thermal properties. They are high strength, resistant against aggressive chemicals and all common acids and bases, extremely heat resistant and noncombustible. Technically they are used, e.g., in bulletproof vests, heat resistant clothing for firefighters and soldiers or as reinforcement fibers in composites used in airplanes and cars [1,2].

For most applications of m-aramids, customers request the dyeing of the material. However, the conventional dyeing of aramids is an extremely difficult, time-consuming and costly process. In particular, it needs polluting chemicals, the so-called carriers, which allow the dyestuff to penetrate the fiber matrix. Such compounds, e.g., benzyl alcohol, benzaldehyde, acetophenone and, in some countries, even chloro-organic compounds, are highly controversial in view of workplace exposure and environmental impact [3,4]. Therefore, strict regulations for the use and the appropriate disposal of the dyeing liquors exist in many countries. To meet these requirements, the dyeing companies already face a huge technical and ecological effort, which significantly raises the overall costs for aramid manufacturing. In the medium term, governments, e.g., in the European Union, plan the further decrease in limit values or even an entire ban on such carriers, which—in default of alternatives—could endanger many jobs in this textile sector.

Thus, textile industries around the world have, for many years, been looking for alternative dyeing techniques for aramid fibers—in particular to avoid the discussed use of the problematic carriers.

In the past, scientists have reported the use of ionic liquids (IL) in various textile-related processes, e.g., for textile finishing, textile functionalization and for the spinning of textile fibers, as well as for the dyeing of textiles [5]. Ionic liquids—often referred to as “green solvents”—are salts with a melting point lower than 100 °C. ILs excel by their extremely low vapor pressure, high thermo-stability and nonflammability, which makes them easy to handle in typical textile finishing processes. Moreover, ILs have high and temperature-related dielectric constants, therefore exhibiting outstanding solvent power for various textile-related substances, such as cellulose, keratin and silicones. Like common salts, ILs consist of cations and anions, e.g., imidazolium, pyridinium, phosphonium plus ammonium (cations) and alkyl sulfates, hexafluoro phosphate and tetrafluoro borate plus halogenides (anions). These two components can be varied in a broad range, yielding designed properties. For instance, melting point, viscosity, density and hydrophobicity can be adjusted for specific applications. In the non-textile sector, ionic liquids have already reached the market. Examples are solvents (catalysis, organic syntheses, polymerizations, synthesis of nanoparticles), electrolytes (batteries, fuel cells, sensors), separation techniques (membranes, gas separation, extractions), analytics (gas chromatography, protein crystallization), lubricants and fuel additives [6,7,8].

We published our first study on the IL-based dyeing of common fiber types, such as cotton and polyester, in 2006 [9,10]. Yuan et al. (2010) used 1-butyl-3-methylimidazolium chloride to increase the dye uptake in wool colorization [11]. Additionally, 1-(2-hydroxyethyl)-3-methylimidazolium chloride, 2-hydroxidiethanolamine and a glycerine-based eutectic solvent were employed to increase, e.g., the color strength of polyester textiles [12,13,14]. In our own study, we have successfully demonstrated that polyethylene terephthalate (PET) fibers can be dyed in manifold shades with commercial disperse dyestuffs in various commercial ILs far above 100 °C, using open non-pressurized systems. Compared to conventionally dyed PET, the fibrous material shows no significant differences in terms of mechanical properties and fastness to rubbing, washing and light. In addition, we have successfully demonstrated that the typical auxiliaries of commercial dyestuffs are not necessary within the IL dyeing procedure. Furthermore, we have shown that PET/cotton blends can generally be dyed in one step using both needed dyestuff types in one dyeing liquor (mixture of reactive dyes for cotton and disperse dyes for PET) [15].

In widening our general IL dyeing concept, here, we present a study on the dyeing of the high-performance m-aramid fiber with ionic liquids in the absence of any polluting carrier. In the first step, the dyeing quality and the influence of fiber crystallinity were studied for different classes of dyestuffs. For two chosen systems, the relevant physical chemical dyeing parameters, such as tensile strength, elongation and fastness to washing, rubbing and light, were investigated systematically.

## 2. Materials and Methods

### 2.1. Materials

The experiments were carried out using the amorphous m-aramid fabric Nomex Comfort (surface weight 245 g/m^2^) and the crystalline m-aramid fabric Nomex 450 (surface weight 140 g/m^2^). For the comparison of the light fastness, we used conventionally dyed Nomex Comfort as a reference material (surface weight 245 g/m^2^, dyed blue, black, orange with the use of carriers). All textiles were purchased from IBENA Textilwerke GmbH (Bocholt, Germany). For the dyeing experiments, we used several different ionic liquids. Most of the discussed experiments were carried out with 1-ethyl-3-methylimidazolium ethyl sulfate (EMIM-EtSO_4_, Sigma-Aldrich, St. Louis, MO, USA). Commercial cationic dyestuffs were used for the dyeing of the aramid fibers. Doracryl^®^ Red GL, Yellow XGRL, Blue GL and Orange R were purchased from M. Dohmen GmbH (Moenchengladbach, Germany). The acid dyestuff Nigrosin (Acid Black 2) was obtained from Sigma-Aldrich. The pure fluorescent dyestuffs for PET Maxilon^®^ Flavine crude were purchased from Huntsman (Langweid a.L., Germany).

### 2.2. General Dyeing Procedure

Three hundred micrograms of the respective dyestuffs were dissolved in 15 g EMIM-EtSO_4_ (or other IL). A piece of aramid fabric (2 cm × 2 cm) was submersed in the preheated solution and kept there for one hour at the specified temperature without stirring. After the dwell time, the fabric was removed from the dyeing bath and was put into a beaker with water. Afterwards, the fabric was rinsed with running water till the water was clear. Then the fabric was dried overnight at room temperature. All colored textile materials were additionally rinsed with acetone to remove excessive dyestuffs.

### 2.3. Characterization

UV–Vis spectra of the samples were evaluated using a Cary 5E spectrophotometer (Varian GmbH, Waldbronn, Germany). The colorimetric measurements were conducted by a Colorlite sph850 (Colorlite GmbH, Katlenburg-Lindau, Germany) using L*a*b* color space. The mentioned color distances ΔE are the differences between the uncolored and the colored textile materials. ΔE is calculated as $\Delta E\;=\;\sqrt{\Delta L^{2}\;+\;\Delta a^{2}\;+\;\Delta b^{2}}$. To evaluate fiber cross sections of the dyed textile material, single fiber filaments were removed from the fabric and embedded in instant adhesive between two polypropylene plates. Thin slices of the cured sample were cut off and put on object plates. The samples were examined by microscopy using a VHX digital microscope (Keyence Deutschland GmbH, Neu-Isenburg, Germany). Scanning electron microscopy of the fibers was conducted with a SEM S-3400 N II (Hitachi High-Technologies Europe GmbH, Krefeld, Germany). Mechanical properties of the m-aramid fabric were characterized by the maximum force and elongation at maximum force measured according to DIN EN ISO 13934-1 using a ZMART pro Typ 1455 (Zwick GmbH & Co KG, Ulm, Germany) (standard atmosphere: 20 °C, 65% rel. humidity, clamping length: 200 mm, fabric width: 30 mm, draw rate: 100 mm/min, preload force: 5 N). The fastness to washing (scale 1–5, best achievable level 5) was conducted according to DIN EN ISO 105-C06 using a Linitest plus (Atlas Material Testing Technology GmbH, Linsengericht-Altenhasslau, Germany). The light fastness (scale 1–8, best achievable level 8) was evaluated according to DIN EN ISO 105-B02 using a Xenotest 150 S (Atlas Material Testing Technology GmbH, Altenhasslau, Germany). The fastness to rubbing (scale 1–5, best achievable level 5) was conducted according to EN ISO 105-X12 using a Crockmeter (Crockmaster, James Heal, UK) (standard atmosphere: 20 °C, 65% rel. humidity, conditioning: 4 h, cone for rubbing: cylindrical (16 mm), weight of 9 N, absorption of water: 95–100%).

## 3. Results

### 3.1. Preselection of Ionic Liquid

**Figure 1:**
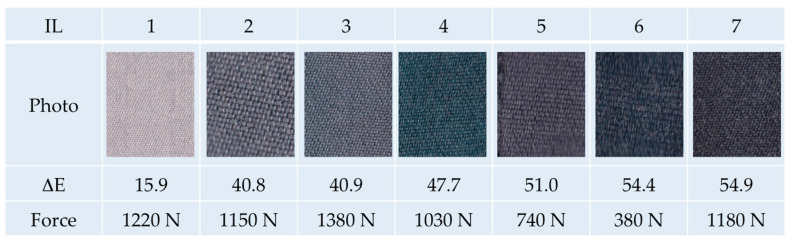
Dyeing of amorphous m-aramid fabrics with Acid Black 194 in various ionic liquids at 180 °C (photo, color distance and tensile strength, 1 = Tis(2-hydroxyethyl)methylammoniumethylsulfate, 2 = BMIM-triflate, 3 = EMIM-triflate, 4 = EMIM-dicyanamide, 5 = Tributylmethylphosphonium methylsulfate, 6 = EMIM-methanesulfonate, 7 = EMIM-EtSO_4_, tensile strength blank fabric = 1090 N).

In general, m-aramid is known as a highly thermo-stable polymer. However, the solving power of certain ILs at high temperatures may affect the polymer’s stability, yielding in a loss of tensile strength of the treated fabrics. Therefore, we started our investigations with a pre-selection of the most suitable ILs for optimum dyeing result and minimum fiber damage. Figure 1 summarizes our first dyeing experiments of amorphous aramid fabrics using various ILs and the acid dyestuff Acid Black 194 at 180 °C.

**Figure 2:**
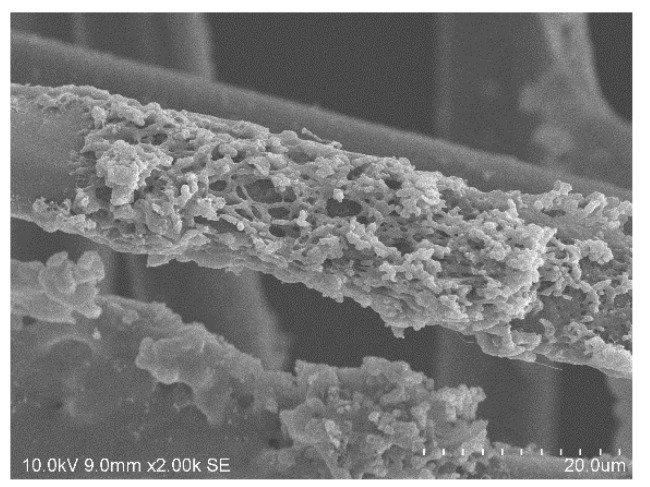
SEM micrograph of amorphous m-aramid fibers in EMIM-Ac with Acid Black 194 at 180 °C.

The photographs and the corresponding color distances document that only IL 5, 6 and 7 yield a promising dyeing result. In contrast, e.g., IL 1 gives a ΔE of only 15.9, indicating that the IL is not able to penetrate the fiber matrix. On the other hand, IL 5 and 6 have a significant impact on the fiber´s stability, excluding them from further investigations. Exemplarily, Figure 2 shows a SEM micrograph of an extremely destroyed fiber after the dyeing procedure with the IL EMIM-acetate. From this fiber and fabric, we were not able to measure the color distance or the tensile strength.

Only IL 7 (EMIM-EtSO_4_) shows both a good dyeing result and the conservation of the physical properties. Thus, we decided at a very early stage to conduct all further experiments with IL EMIM-EtSO_4_. Beside the promising screening results, EMIM-EtSO_4_ excels at its inherent properties, such as being liquid at room temperature, benign in view of water pollution potential and without strict safety regulations, all of which promise technical and industrial applicability.

### 3.2. Dyeing of m-Aramid Fibers in EMIN-EtSO_4_

**Figure 3:**
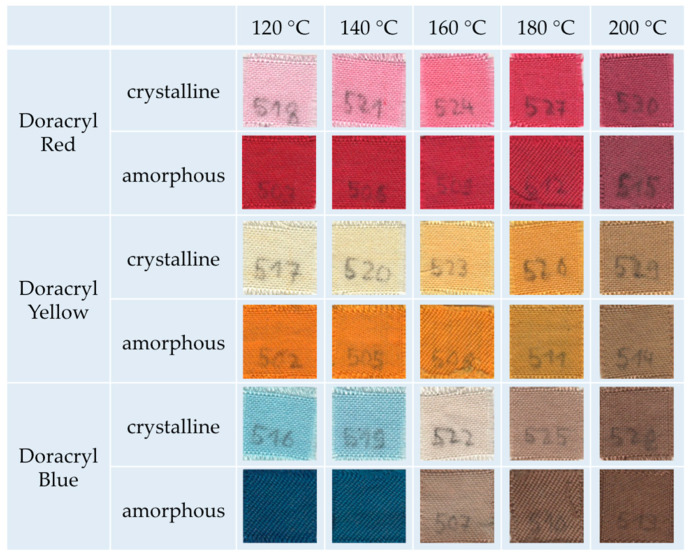
Temperature-dependent dyeing of amorphous and crystalline m-aramid fabrics with various cationic dyes in EMIM-EtSO_4_.

We go on with the dyeing of amorphous and crystalline m-aramid fibers in the IL EMIM-EtSO_4_. The latter species is very difficult to dye in conventional procedures. Figure 3 shows photographs of the dyeing results using three kinds of typical cationic dyestuffs used in aramid dyeing.

**Figure 4:**
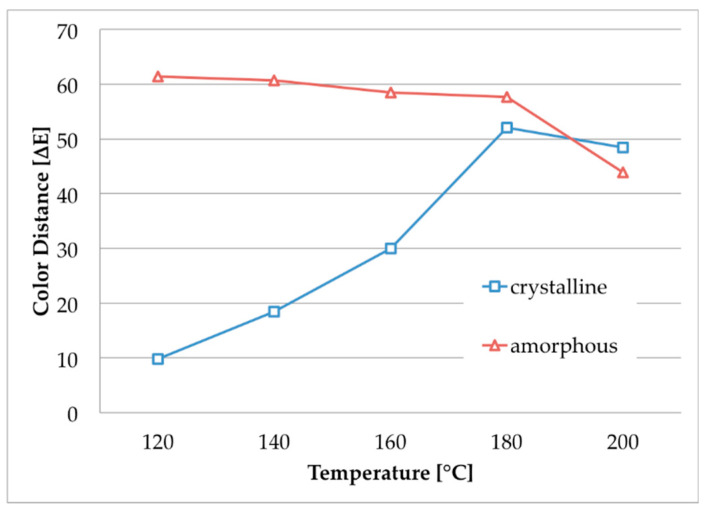
Color distances of amorphous and crystalline m-aramid fabrics after dyeing with Doracryl Red in EMIM-EtSO_4_ (temperature range 120–200 °C, for pictures see Figure 3, row 1 and 2).

Generally, the results demonstrate that the amorphous m-aramid fibers can be dyed, leading to excellent color shades for all investigated dyestuffs in the temperature range of 120–140 °C. In addition, even a dyestuff penetration was observed for the crystalline m-aramid fibers at higher temperatures. In the case of Doracryl Red, nearly the same dyeing result at 180 °C was obtained for both the amorphous and the crystalline specimen, as can be seen in the measurement of the corresponding color distances (Figure 4).

**Figure 5:**
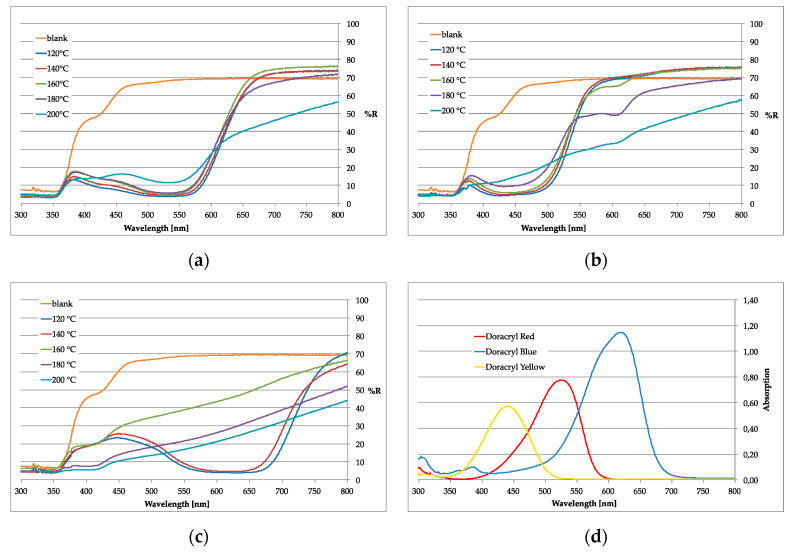
UV–Vis remission spectra of aramid fabrics after dyeing with various cationic dyestuffs in EMIM-EtSO_4_ at different temperatures; (**a**) Doracryl Red; (**b**) Doracryl Yellow; (**c**) Doracryl Blue; (**d**) UV–Vis absorption spectra of the pure dyestuffs.

However, as suggested by the dyeing results shown in Figure 3, a dyestuff-specific degeneration of the dyestuff itself occurs at higher temperatures. Figure 5 shows the UV–Vis spectra of the amorphous textiles after dyeing at certain temperatures. While Doracryl Red (Figure 5a) is thermo-stable even at 180 °C, a fading of Doracryl Yellow (Figure 5b) between 160 °C and 180 °C was observed. Doracryl Blue (Figure 5c) degenerated above 140 °C. For a better comparison, Figure 5d shows the absorption spectra of the pure dyestuffs. As a first result, the dyeing procedure for our innovative dyeing process in IL should be executed in the temperature range of 120–140 °C, at which temperature typical cationic dyestuffs do not lose their color brightness.

**Figure 6:**
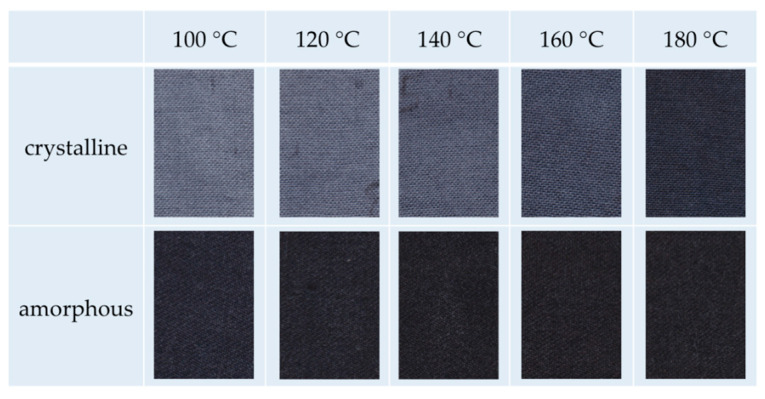
Temperature-dependent dyeing of amorphous and crystalline m-aramid fabrics with Nigrosin in EMIM-EtSO_4_.

**Figure 7:**
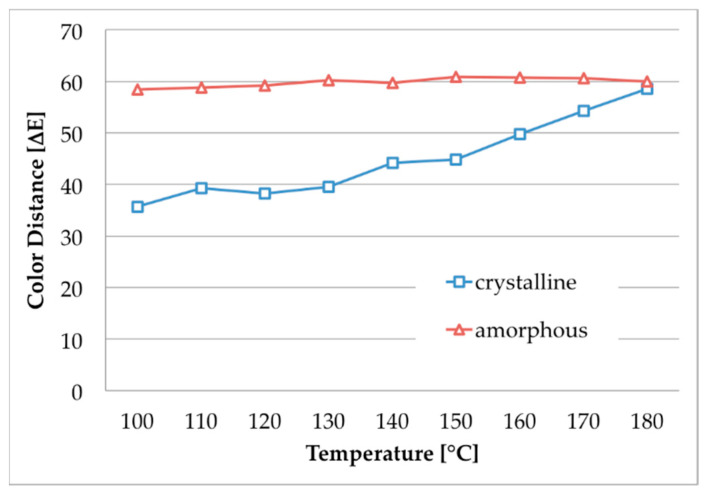
Color distances of amorphous and crystalline m-aramid fabrics after dyeing with Nigrosin in EMIM-EtSO_4_ (temperature range 100–180 °C).

Acid dyes are known to be more thermo-stable than cationic dyes. Therefore, we carried out some experiments with the acid dyestuff Nigrosin to compare the dyeing result of amorphous and crystalline m-aramid fabrics at dyeing temperatures up to 200 °C, where a sufficient fiber penetration was assumed even in the crystalline polymer. The photographs in Figure 6 and the corresponding color distances in Figure 7 indicate that the difference in color shades of both species decreases and finally vanishes with rising dyeing temperature. While a perfect dyeing was observed for the amorphous material at 100 °C, nearly the same outstanding result can be generated for the crystalline fabric at 180 °C.

**Figure 8:**
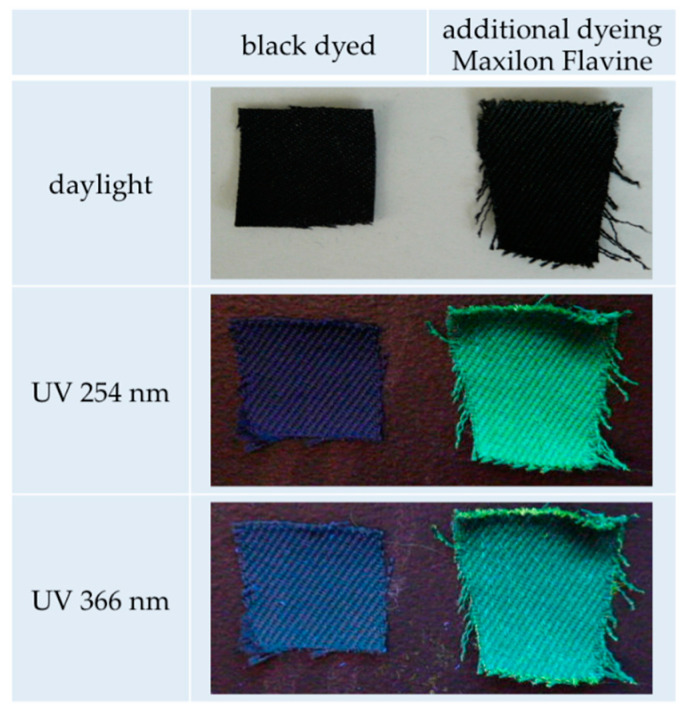
Fluorescent properties of a pre-dyed m-aramid fabric after additional dyeing with a fluorescent dyestuff in EMIM-EtSO_4_.

In a side study, we also investigated whether our technology is useful for special and highly requested effects, such as fluorescent properties, which can be used, e.g., in the production of safety jackets. Figure 8 shows photographs taken in daylight and under two different UV wavelengths. The left fabric was dyed black, while the right was dyed black and subsequently over-dyed with a fluorescent dyestuff in EMIM-EtSO_4_. While the fabrics appear identical in normal light, a pretty nice green fluorescent behavior was observed from the over-dyed sample under UV irradiation.

### 3.3. Properties of Amorphous m-Aramid Fibers Dyed in EMIN-EtSO_4_

Having shown that the dyeing of m-aramid fibers generally works in IL, it was essential to consider all textile-related properties that may be of interest for processing and application. All further experiments were conducted using an amorphous m-aramid fabric. We compare the results using EMIN-EtSO_4_ and the cationic dyestuff Doracryl Orange and the acid dyestuff Nigrosin at 120 °C and 180 °C, respectively.

**Figure 9:**
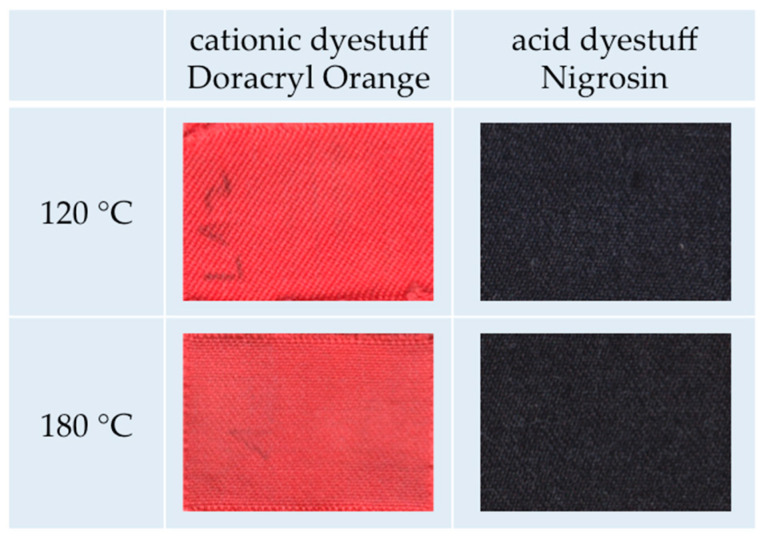
Dyeing of amorphous m-aramid fabrics with cationic dyestuff Doracryl Orange and acid dyestuff Nigrosin in EMIM-EtSO_4_ at 120 °C and 180 °C, respectively.

First, Figure 9 shows photographs of the dyed fabrics. All dyed specimens exhibit a brilliant color shade comparable to conventionally dyed aramid textiles (it should be noted that Doracryl Orange is thermo-stable at 180 °C).

**Figure 10:**
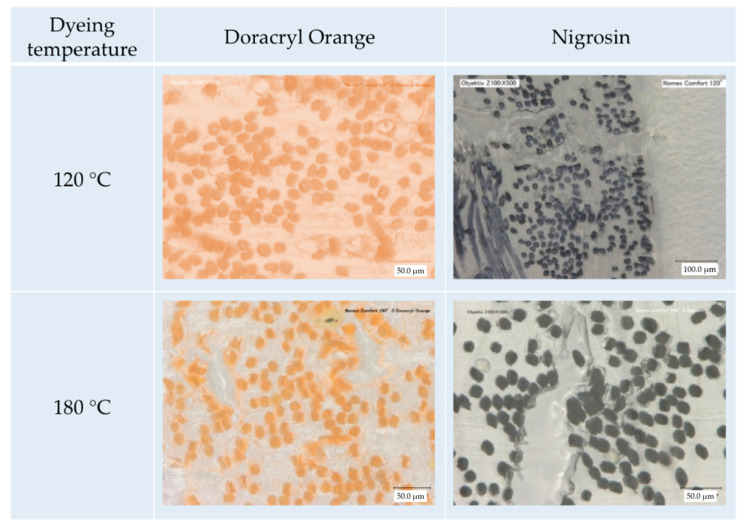
Fiber cross sections of amorphous m-aramid fabrics dyed with cationic dyestuff Doracryl Orange and acid dyestuff Nigrosin in EMIM-EtSO_4_ at 120 °C and 180 °C, respectively.

**Figure 11:**
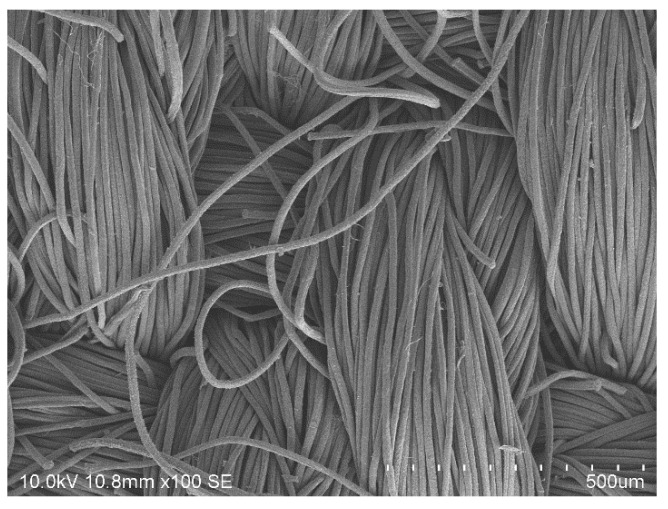
SEM micrograph of amorphous m-aramid fibers dyed with Nigrosin in EMIM-EtSO_4_ at 180 °C.

The photographs of fiber cross sections give a hint as to whether the dyestuff is only penetrating the outer surface region (resulting often in an insufficient washing resistance) or the full fiber cross section. The cross sections shown in Figure 10 indicate that a deep and full saturation is achieved for both dyestuffs at 120 °C. SEM analysis (an exemplary micrograph is shown in Figure 11) further reveals that no fiber damage occurs.

As a quantitative measure of effect on mechanical properties or even fiber damage, we measured the corresponding tensile strengths of the textiles dyed in IL, shown in Table 1. We detect a slight influence on the fiber stability after dyeing at 120 °C. The tensile strengths decrease about 7% compared to the blank material, while the average elongation at break slightly increases. In contrast, the fabrics dyed at 180 °C show significantly better mechanical properties. This could be a recrystallinization process at that high temperature, but a full clarification is only possible by, e.g., X-ray crystallography, which will be part of our future R&D work.

**Figure 12:**
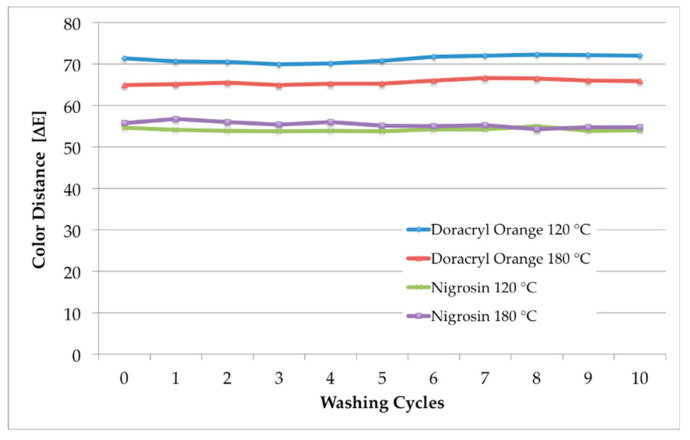
Color distances of amorphous m-aramid fabrics dyed in EMIM-EtSO_4_ with Doracryl Orange and Nigrosin at 120 °C and 180 °C, respectively, after 1, 5 and 10 washing cycles.

Table 2 summarizes the results of the wash tests. As already expected from the determination of the fiber cross sections (Figure 10), we found an outstanding washing fastness. All EMIM-EtSO_4_-dyed textiles exhibit the best or nearly the best level of 5, which was additionally documented by the measurement of the corresponding color distances shown in Figure 12. No significant change in color shade was observed even after 10 washing cycles.

From the industrial point of view, the light fastness of dyed m-aramid fibers is one of the most challenging issues. As shown in Table 3, conventionally dyed m-aramids often show a low level of light fastness, in the range of 2–4. A grade of 5 is only achieved if an additional fluoro-carbon (FC) finishing is conducted. The materials dyed in IL show at least comparable or even significantly better results up to a level of 6 (Table 4), which should attract the special interest of the aramid dyeing and finishing industry.

Finally, we checked the fastness to rubbing of the IL-dyed textiles. All textile specimens show the best or nearly the best level of 5 (Table 5). The outstanding fastness results to washing, light and rubbing are in line with the deep fiber penetration of the dyestuff proven by the above-mentioned fiber cross sections (see Figure 10). The dyestuff is well protected from mechanical and physical stress by the surrounding fiber matrix.

## 4. Discussion

High-performance textiles made of aramid fibers supply a huge and steadily growing market of important safety products and light-weight materials, where light but strong and thermo-stable materials are desired. A long-standing problem in this field, however, is the conventional dyeing procedure of aramid fibers, which does not meet modern requirements for employment protection and pollution control. With our new operational dyeing technique using ILs, we have successfully demonstrated that the coloration of m-aramids is possible in the absence of any carriers, the widely used but most problematic auxiliaries in aramid dyeing.

Our main findings are:

- the dyeability of m-aramid fibers strongly depends on their crystallinity;
- amorphous m-aramid fibers are generally dyeable in ILs with typical cationic dyes;
- EMIM-EtSO_4_ represents the most promising IL, yielding bright and uniform shades;
- the recommended temperature is 120–140 °C;
- m-aramid fibers with higher crystallinity can be dyed with thermo-stable dyestuffs at higher temperatures, e.g., acid dyes;
- no (ill) effect on the mechanical properties;
- full fiber penetration;
- excellent fastness towards washing, rubbing and light;
- no harmful and polluting carriers are needed.

The results are in line with our own findings on the dyeing of other fiber types, such as polyester, cotton and their blends in ILs. In all cases, deep-colored textiles could be obtained with outstanding textile-related properties. For both the dyeing of polyester and the dyeing of m-aramids, the dyeing results are comparable or even better than conventionally dyed textiles. Thus, by simply changing the used IL and the fiber-specific dyestuff type, a comparable dyeing procedure was successfully implemented for a high-performance fiber for the first time. Therefore, this study represents a consequent continuation of our previous work, as well as the approaches of other authors.

## 5. Conclusions

Today, ionic liquids, as so-called *green solvents*, play an important role in worldwide research and some technical applications are already being put into practice. Several studies are dealing with the use of ILs even in textile-related processes, such as fiber finishing, functionalization or fiber spinning. In particular, the dyeing of synthetic, as well as natural, fibers in ILs is a growing research objective and some remarkable achievements in this field have been obtained. However, ILs are not yet established commercially in the textile dyeing sector since the existing dyeing technologies for, e.g., polyester or cotton, have not been called into question to date. This is different in the case of the high-performance fiber m-aramid, where the conventional dyeing procedure has huge economic and ecological disadvantages. Our new fundamental and application-oriented study adds to the knowledge on the use of ionic liquids in general and could help to overcome one of the most challenging issues in the dyeing of m-aramid fibers, the relinquishment of carrier chemicals, which will probably be banned within the next few years in many countries worldwide. However, further R&D is needed to industrialize our concept. Our future work will focus on, from an economic point of view, the fundamentally needed recovery of the IL itself by, e.g., ultra-nano filtration of the dyeing liquor.

## Figures and Tables

**Table 1:** Mechanical properties of amorphous m-aramid fabrics after dyeing in EMIM-EtSO_4_ with Doracryl Orange and Nigrosin at 120 °C and 180 °C, respectively.

Dyestuff	Dyeing Temperature	Average Tensile Strength	Average Elongation at Break
-	-	798 N	31.9%
Doracryl Orange	120 °C	734 N	32.9%
	180 °C	907 N	36.6%
Nigrosin	120 °C	740 N	33.8%
	180 °C	900 N	37.3%

**Table 2:** Fastness to washing of amorphous m-aramid fabrics dyed in EMIM-EtSO_4_ with Doracryl Orange and Nigrosin at 120 °C and 180 °C, respectively, after 1, 5 and 10 washing cycles (accompanying fabric cotton/polyamide).

Number of Washing Cycles	Doracryl Orange		Nigrosin	
	120 °C	180 °C	120 °C	180 °C
1	5/5	4.5/4.5	5/5	4.5/4.5
5	5/4.5	5/5	4.5/4.5	5/5
10	5/5	5/5	4.5/4.5	5/4.5

**Table 3:** Fastness to light of conventionally dyed m-aramid fibers.

Reference Material	Color	Fastness to Light
Nomex Comfort	blue	2–3
Nomex Comfort	black	3–4
Nomex Comfort FC finished	orange	5

**Table 4:** Fastness to light of amorphous m-aramid fabrics dyed in EMIM-EtSO_4_ with Doracryl Orange and Nigrosin at 120 °C and 180 ° C, respectively.

Dyestuff	Dyeing Temperature	
	120 °C	180 °C
Doracryl Orange	4	4
Nigrosin	4–5	6

**Table 5:** Fastness to rubbing of amorphous m-aramid fabrics dyed in EMIM-EtSO_4_ with Doracryl Orange and Nigrosin at 120 °C and 180 ° C, respectively.

Dyestuff	Dyeing Temperature	Dry	Wet
Doracryl Orange	120 °C	4–5	4–5
	180 °C	4–5	4–5
Nigrosin	120 °C	4	4–5
	180 °C	4–5	4–5

## References

[1] Lawrence C.A. (2014). High Performance Fibers and Their Applications.

[2] Reglero Ruiz J.A., Trigo-López M., García F.C., García J.M. (2017). Functional Aromatic Polyamides. Polymers.

[3] Nechtawal A., Rossbach V. (1999). The carrier effect in the m-aramid fiber/cationic dye/benzyl alcohol system. Text. Res. J..

[4] Zheng H.-D., Zhang J., Yan J., Zheng L.-J. (2017). Investigations on the effect of carriers on meta-aramid fabric dyeing properties in supercritical carbon dioxide. RSC Adv..

[5] Meksi N., Moussa A. (2017). A review of progress in the ecological application of ionic liquids in textile processes. J. Clean. Prod..

[6] Kirchner B. (2010). Ionic Liquids.

[7] Mac Farland D.R., Kar M., Pringle J.M. (2017). Fundamentals of Ionic Liquids: From Chemistry to Applications.

[8] Fehrmann R., Santini C. (2019). Ionic Liquids, Synthesis Properties, Technologies and Applications.

[9] Knittel D., Schollmeyer E. (2015). Dyeing Technique for Synthetic and Cellulosic Fibers Using Ionic Liquids. German Patent.

[10] Knittel D., Schollmeyer E. (2007). Ionic Liquids for textile finishing, part 1: Dyeing of textiles. Melliand Text..

[11] Yuan J., Wang Q., Fan X., Wang P. (2010). Enhancing dye adsorption of wool fibers with 1-butyl-3-methylimidazolium chloride ionic liquid processing. Text. Res. J..

[12] Bianchini R., Cevasco G., Chiappe C., Pomelli C.S., Rodriguez Douton M.J. (2015). Ionic liquids can significantly improve textile dyeing: An innovative application assuring economic and environmental benefits. ACS Sustain. Chem..

[13] Andrade R.S., Torres D., Ribeiro F.R., Chiari-Andreéo B.G., Oshiro J.A., Iglesias M. (2017). Sustainable cotton dyeing in nonaqueous medium applying protic ionic liquids. ACS Sustain. Chem. Eng..

[14] Pawar S.S., Maiti S., Biranje S., Kulkarni K., Adivarekar R.V. (2019). A novel green approach for dyeing polyester using glycerine based eutectic solvent as a dyeing medium. Heliyon.

[15] Opwis K., Benken R., Knittel D., Gutmann J.S. (2017). Dyeing of PET fibers in ionic liquids. Int. J. New Technol. Res..

